# Overview of Bovine Mastitis: Application of Metabolomics in Screening Its Predictive and Diagnostic Biomarkers

**DOI:** 10.3390/ani14152264

**Published:** 2024-08-04

**Authors:** Muyang Li, Zhongjie Li, Ming Deng, Dewu Liu, Baoli Sun, Jianying Liu, Jianchao Guo, Yongqing Guo

**Affiliations:** 1 College of Animal Science, South China Agricultural University, Guangzhou 510642, China; 15267561605@163.com (M.L.); 17613779127@163.com (Z.L.); dengming@scau.edu.cn (M.D.); dwliu@scau.edu.cn (D.L.); baolisun@scau.edu.cn (B.S.); 2 Agro-Tech Center of Guangdong Province, Guangzhou 510500, China; ljy1457@163.com

**Keywords:** metabolomics, bovine, mastitis, diagnosis, prediction, biomarkers

## Abstract

**Simple Summary:**

This review outlines the use of metabolomics to detect biomarkers for bovine mastitis, offering insights into disease prediction and early diagnosis. It details the use of various biological samples for metabolomic analysis, including milk, blood, urine, rumen fluid, feces, and mammary tissue.

**Abstract:**

Bovine mastitis is an inflammatory disease of the mammary glands, and its pathogenesis and diagnosis are complicated. Through qualitative and quantitative analysis of small-molecule metabolites, the metabolomics technique plays an important role in finding biomarkers and studying the metabolic mechanism of bovine mastitis. Therefore, this paper reviews the predictive and diagnostic biomarkers of bovine mastitis that have been identified using metabolomics techniques and that are present in samples such as milk, blood, urine, rumen fluid, feces, and mammary tissue. In addition, the metabolic pathways of mastitis-related biomarkers in milk and blood were analyzed; it was found that the tricarboxylic acid (TCA) cycle was the most significant (FDR = 0.0015767) pathway in milk fluid, and glyoxylate and dicarboxylate metabolism was the most significant (FDR = 0.0081994) pathway in blood. The purpose of this review is to provide useful information for the prediction and early diagnosis of bovine mastitis.

## 1. Introduction

Mastitis is a multifactorial and complex mammary inflammatory disease caused by the interaction of host, environment and typical bacteria [1,2]. The incidence of mastitis increases during the periparturient period [3], and mastitis is one of the most common and challenging diseases in dairy cow production. It is categorized as environmental or contagious by origin and clinical (CM) or subclinical (SCM) by symptoms [4]. CM features obvious symptoms of inflammation in the mammary gland and milk, redness and swelling of the mammary gland, and milk abnormalities including flaky and coagulated foreign substances [5]. SCM is the most common type of mastitis and has no obvious symptoms [6]. CM is further divided into acute, subacute, and chronic forms, with acute cases being sudden and severe, and chronic ones characterized by duration or recurrence [7]. Mastitis not only causes a decrease in milk production and quality, but also increases the treatment and herd replacement costs, which is detrimental to animal welfare and leads to food safety issues [8]. In the United States, the annual economic cost of mastitis is about USD 2 billion [9]. The cost of each case of mastitis varies widely, from USD 112.2 to USD 315.7 [10], and the loss due to decreased milk production accounts for 70% of the total loss [11,12]. The average loss of milk production in 305 days per cow is about 200 L [12]. Another study, based on a survey of dairy producers and the development of economic models, estimated that Canadian dairy farms spent CAD 662 per cow in one year to treat or prevent mastitis [13].

The pathogenesis and diagnostic methods of mastitis are very complex. Traditionally, SCC and bacterial culture are commonly used to determine mastitis. In recent years, the continuous development of new technologies has made the search for biomarkers of this disease a major research craze [14]. Biomarkers are indicators to detect physiological processes and pathological states in the body. According to their uses, biomarkers can be categorized into diagnostic, monitoring, and predictive, each serving a pivotal role in disease diagnosis, drug development, and disease prevention and treatment [15]. The omics technique is widely used in cow disease research, and metabolomics can systematically reveal the dynamic changes in metabolites molecules, as well as help us comprehensively understand the metabolic changes caused by diseases in animal body fluids and tissues at the molecular level.

**Figure 1:**
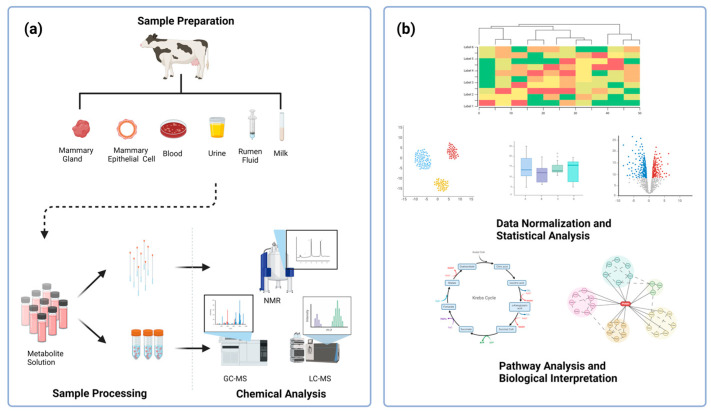
A technical roadmap for the metabolomics technique. The metabolomics technique, regardless of the experimental platform, can be divided into five processes, including (**a**) sample preparation, sample processing (metabolite extraction and derivatization), chemical analysis (compound identification via platforms, such as NMR, GC−MS, LC−MS), (**b**) data normalization and statistical analysis, and pathway analysis and biological interpretation. Created with BioRender.com.

Metabolomics consists in the qualitative and quantitative analysis of small-molecule metabolites in urine, blood, milk, saliva, cells, tissues, feces, and other samples through a variety of analytical methods. Its research objects are generally endogenous small-molecule metabolites with a relative molecular mass of less than 1500 Da [16], including lipids, amino acids, peptides, nucleic acids, organic acids, vitamins, mercaptan, carbohydrate, and so on [17]; it is considered an effective tool for the comprehensive characterization of the small-molecule metabolites present in complex biological systems. The application scope of metabolomics is extensive, extending into the related fields of human, animal, plant, and microbial research. It encompasses disciplines such as toxicology [18], nutriology [19], food science [20], environmental science [21], and many others, demonstrating its broad utility in the study of biological systems. The metabolomics techniques include nuclear magnetic resonance (NMR), gas chromatography–mass spectrometry (GC-MS), and liquid chromatography–mass spectrometry (LC-MS). Among them, the main advantages of GC-MS and LC-MS are their high sensitivity and strong specificity; they are widely used in the analysis of chemical substances in complex substrates. NMR also has unique advantages, such as a relatively simple sample preparation process, fast and unbiased results, easy automation, and high repeatability [22]. As an emerging discipline, metabolomics has the characteristics of high throughput and comprehensibility. The technical route is shown in Figure 1. However, most metabolomics techniques were found to have two limitations. First, because of certain characteristics of metabolites such as large quantity, various physical and chemical properties, wide concentration range, and dynamic changes, it is difficult to accurately determine the whole metabolome in one analysis. The second limitation is the large amount of metabolome data, whose comprehensive management depends on the continuous improvement of technology, calculation, and analysis methods [23]. Therefore, in order to effectively utilize metabolomics in the prevention and treatment of bovine mastitis, it is necessary to comprehensively apply multiple analysis platforms and methods under limited conditions to obtain a more complete metabolic profile and accurately identify metabolites.

Currently, most research on bovine mastitis focuses on genomics, transcriptomics, and proteomics. With the continuous improvement of metabolomics techniques, more and more studies have begun to apply this technology to the analysis of biological fluids and tissues, making it possible to screen the biomarkers of bovine mastitis and providing a basis for researchers to understand the overall metabolic mechanism of bovine mastitis. The first metabolomic study of mastitis was conducted in 2005 and demonstrated through GC-MS that it was possible to distinguish normal and mastitis milk samples using a chemical method known as electronic nose [24]. The metabolites in biological fluids such as milk, blood, urine, rumen fluid, feces, mammary tissue, or mammary epithelial cells can be used as indicators to reflect the mammary gland health status in dairy cows and as biomarkers for the diagnosis of mastitis [25].

Through qualitative and quantitative analysis of small-molecule metabolites, the metabolomics technique has great potential in screening biomarkers of bovine mastitis and studying mastitis pathogenesis. In this paper, the application of the metabolomics technique in biomarker screening for bovine mastitis was reviewed in order to provide a theoretical basis for the prediction and early diagnosis of bovine mastitis and the development of new therapeutic drugs and reduce the economic loss caused by mastitis in dairy cows.

## 2. Methodology

In May 2024, scientific articles in the Web of Science were retrieved. The following search terms were used: “mastitis”, “metabonomics/metabolomics”, “diagnosis”, “pathogenesis”, “prediction”, and “biomarkers”. The literature review focused on the application of metabolomics in the screening and diagnosis of mastitis. For this reason, a total of 81 relevant documents were retrieved with the keywords “mastitis”, “metabonomics/metabolomics”. Inclusion criteria: (1) studies conducted in dairy cows; (2) the application of metabolomics in the screening and diagnosis of mastitis. Exclusion criteria: (1) non-metabolomics studies; (2) metabolomics studies conducted in mice or humans; (3) studies discussing treatment plans for mastitis in dairy cows; (4) studies with unclear or low-quality results; (5) documents with similar or duplicate content. Ultimately, 19 documents were included.

## 3. Pathogenesis of Bovine Mastitis

In order to develop techniques suitable for the diagnosis of mastitis, it is important to explore its pathogenesis [26]. This section briefly summarizes the pathogenic factors of mastitis, as well as summarizes and analyzes the pathogenic mechanisms of common pathogens based on innate and adaptive immune responses to help understand the pathogenesis of bovine mastitis and facilitate its prevention and treatment.

Many pathogenic factors can cause bovine mastitis, among which pathogenic microorganism infection is the main one. There are about 150 kinds of pathogenic microorganisms associated with bovine mastitis [27], including bacteria, fungi, mycoplasma, and viruses. The development of mastitis is related to the degree of mammary gland exposure to bacterial pathogens, and the expression of bacterial virulence factors can affect the occurrence and severity of infection [28]. A wide range of Gram-positive and Gram-negative pathogens can cause mastitis. A survey on the prevalence of mastitis in Ethiopia from 2005 to 2022 showed that 84.70% of mastitis was caused by Gram-positive bacteria, and 15.30% by Gram-negative bacteria [29]. Bovine mastitis is mostly caused by *Staphylococcus*, *Streptococcus*, and *Enterobacter* bacteria, which can be infectious and contagious (e.g., *Staphylococcus aureus*, *Streptococcus agalactiae*) or environmental (e.g., *Escherichia coli*, *Enterococcus* spp., coagulase-negative *Staphylococcus, Streptococcus uberis*) [30]. *Staphylococcus aureus* can cause CM and SCM, mainly the latter, while *Escherichia coli* often causes CM [31]. Coagulase-negative *Staphylococcus* is weak in pathogenicity and generally cannot cause severe mastitis [32]. *Lactococcus lactis* used as a probiotic was found to cause CM [33]. Compared with mastitis caused by pathogenic bacteria such as *Staphylococcus aureus*, *Streptococcus agalactiae*, and *Escherichia coli*, mastitis induced by *Klebsiella* spp. is not common, but in recent years, the detection of *Klebsiella* spp. in milk samples has sharply increased worldwide [34].

The increased susceptibility and severity of mastitis are also due to a number of host-related factors, including cow self-factors (high parities, high yields), environmental factors (poor hygiene, high heat and humidity, poor ventilation), management factors (incorrect use of the milking equipment, irregular milking procedures), nutritional factors (nutrition imbalances) [35]. Bovine mastitis may also be secondary to certain diseases, such as subacute ruminal acidosis (SARA), hypocalcemia, ketosis, and so on [36]. In addition, it takes two hours for the sphincter muscle to contract and close the teat canal after milking, resulting in the duct remaining dilated and open for a period of time, and this physiological mechanism is an important reason for the increased chance of pathogen invasion [37].

The innate and adaptive immune responses of the mammary gland are complex, interconnected, and critical to blocking mastitis-related pathogens [28]. Among them, the components of the innate immune response include the non-specific barrier effect of the teat canal, pattern recognition receptors (PRRs), phagocytes such as neutrophils and macrophages, soluble factors such as cytokines, complement, and lactoferrin [38]. The innate and adaptive immune defenses of the mammary gland require a high degree of coordination to eliminate sources of tissue damage and restore immune homeostasis.

The teat canal is connected upward to the teat cistern and opens downward at the teat end. The main way for contagious and environmental pathogens to infect the mammary gland is through the teat canal [39]. The papillary sphincter muscle and a keratin layer are important components of the non-specific barrier of the teat canal and play physical and chemical defense roles against invading pathogens. The papillary sphincter muscle consists of longitudinal smooth muscle fibers that surround the lumen of the teat canal; it keeps the teat canal closed during non-lactation periods, preventing milk from flowing out of the mammary gland, and tightly compacts keratin in the teat canal to prevent infection [40]. Keratin is a waxy substance formed by the continuous peeling and shedding of squamous epithelial cells in the inner wall of the teat canal. Its defense against external pathogen invasion is mainly provided in two ways. First, it can accumulate in the lumen of the teat canal and block the lumen; second, it contains esterified and non-esterified fatty acids that can exert antibacterial and bactericidal activities [41].

When the bacteria causing mastitis pass the teat canal barrier, bacterial byproducts (such as lipopolysaccharides (LPSs) or the outer-membrane vesicles (OMVs) of Gram-negative bacteria) act as pathogen-associated molecular patterns (PAMPs) to activate PRRs (including TLRs, NLRs, and RLRs) in macrophages, dendritic cells, and mammary epithelial cells, thereby further activating the NF-κB and MAPK signaling pathways [42]. Then, the inflammatory response is triggered, resulting in increased phagocytic activity of phagocytic cells, increased secretion of antibacterial substances, and PMNs releasing enzymes that damage the local lactating cells, which causes acinous fibrosis and changes in the tissue structure of the affected organ or body cavity [43]. The inflammatory cascade not only leads to elevated local levels of antimicrobial factors, but also may cause damage to the host tissues, leading to the development of acute or chronic mastitis, which adversely affects the production and quality of milk [28]. The MAPK and NF-κB cascades are important inflammatory signaling pathways in mammary gland cells. The phosphorylation of proteins in the MAPK signaling pathway may be an important factor in the occurrence of mastitis, and the activation of the MAPK pathway can also induce the activation of the NF-κB pathway, leading to the occurrence of bovine mastitis [35]. After binding to TLRs on mammary epithelial cells, LPS, a virulence factor of bacteria, causes disease through the NF-κB pathway, in which TLR4, IL-1β, IL-6, TNF-α, and MYD88 are key players [44]. Blocking the NF-κB signaling pathway may be an effective strategy to control mastitis [31]. When the innate immune mechanism fails to eliminate the pathogens, it triggers an adaptive immune response mediated by T and B lymphocytes, which are activated by antigen-presenting cells, MHC II, and various cytokines that play a cytotoxic role, activating B cells to produce antibodies and create a memory for a specific antigen [45,46].

## 4. Diagnosis of Bovine Mastitis

CM can be diagnosed by the abnormal appearance of the udder, such as redness, swelling, and heat, while the two most commonly used methods for identifying SCM in cows are somatic cell count and bacteriological culture. As one of the diagnostic methods for bovine mastitis, SCC has been incorporated into the Dairy Herd Improvement (DHI) program, as it can diagnose mastitis quickly and effectively [47]. At present, SCC in milk is an important parameter in the diagnosis of mastitis, especially SCM [48]. Bovine mastitis may increase the SCC in milk. Most studies showed that the diagnostic error can be minimized when 200,000 cells/mL of milk is used as the threshold for the diagnosis of bovine mastitis. With an SCC > 200,000 cells/mL as the threshold for bovine mastitis, the sensitivity and specificity of identifying the main pathogens of bovine mastitis were 83.4% and 58.9% [49], respectively. An SCC between 100,000 and 200,000 cells/mL indicates that the mammary gland is healthy, but the standard in some countries may be lower than 100,000 cells/mL; SCM could be diagnosed when the SCC increases from 200,000 to 500,000 cells/mL and there are no obvious clinical symptoms. The diagnostic criteria for CM are SCC > 500,000 cells/mL [50]. At present, microscopic counting through methylene blue staining, coulter counting, flow cytometry, and so on is used for SCC determination. However, in the actual production process, in addition to pathogen infection, factors such as season, milking frequency, milking time, parity, and lactation period affect the SCC in milk. Bacterial culture can be performed in a lab or on a farm, and farm culture provides a timelier access to the data [51]. There are two methods for farm bacteriological culture, i.e., the biplate and the triplate systems. The biplate system is based on Gram-positive bacteria selective growth and Gram-negative bacteria selective growth [52]. Compared with the biplate system, the triplate system can help distinguish *Staphylococcus* and *Streptococcus*. Researchers examined the farm biplate system and the triplate system, and the results showed that the biplate system has high specificity but low sensitivity. On the whole, bacteriological culture takes a long time, its labor intensity is relatively large, and the detection costs are relatively high. While most pathogens that can cause mastitis can be grown in medium, some require special growth conditions. Additionally, it is not excluded that the pathogens in a sample are too few to be detected or dead, which leads to false negative results, or that the sample is contaminated by the surrounding environment, which leads to false positive results.

Although SCC and bacteriological culture are considered the golden rule for the diagnosis of dairy mastitis, methods such as PCR, the milk conductivity test, the California Mastitis Test (CMT), and thermal infrared thermography (IRT) are also largely used due to their operating cost, operating time, specificity, and sensitivity. PCR is a molecular diagnostic technique with high sensitivity and specificity. Especially for some bacteria that do not normally grow or grow slowly in medium, PCR shows great effectiveness. With the emergence of multiple PCR-related techniques, such as qPCR and LAMP, the detection of multiple mastitis pathogens at one time was realized [53]. The detection of bovine mastitis by the milk conductivity test is easy to monitor and inexpensive, but relatively insensitive. When inflammation occurs, the concentration of potassium, sodium, and chlorine increases, and the content of lactose decreases, resulting in a change in the conductivity of milk. However, milk conductivity is also affected by age and lactation period. The CMT is simple and fast, but less sensitive, and the results are often misunderstood, especially when diagnosing a case of SCM, as false positive or negative results can be obtained [54]. However, the CMT can identify the main pathogen causing mastitis (*Staphylococcus aureus*, *Streptococcus*, and Gram-negative bacteria), with sensitivity and specificity exceeding 80%. A study found that the body temperature of the diseased part of the cow is slightly higher than normal [54]. Therefore, measuring the temperature by thermal infrared thermography can also determine whether cows have mastitis [55,56]. The advantages and disadvantages of different diagnostic methods for mastitis are shown in Table 1.

Meanwhile, the development of emerging biomarker-assisted technologies for the early detection of inflammatory components and infectious pathogens in mastitis is providing new ideas for the rapid diagnosis and effective prevention of mastitis. Advances in immunoassays, handheld biosensors, nucleic acid testing, enzyme analysis, and genomics, proteomics, and metabolomics have made the early diagnosis of mastitis much easier [57]. With the invention of nanotechnology-based biosensors and the biochip technology, high-throughput analysis using proteomics and metabolomics is now possible [58]. Studies have shown that a combination of several different techniques may lead to better results, and researchers from a variety of fields, including dairy scientists, microbiologists, molecular biologists, biochemists, and nanotechnologists, have joined in the development of methods to detect mastitis pathogens. One example is the combination of real-time polymerase chain reaction with high-resolution melting analysis for the detection of mastitis pathogens. This method can also be used as an alternative to traditional diagnostic methods and offers many benefits, including a low cost and fast results. These studies aim to provide farmers with an integrated approach from sample processing to sample analysis, giving accurate results on the farm and ultimately ensuring animal welfare, improving milk production and quality, and reducing the economic losses in dairy production.

**Table 1:** Comparison of advantages and disadvantages of diagnostic methods for mastitis.

Method	Strengths	Weakness	References
SCC	High sensitivity High specificity	Can be caused by factors other than infection Special equipment required	[59,60]
Bacterial culture	Accurate results Low operational requirements Can be performed on farm	Easily causing milk or medium contamination False positive or negative results present	[61]
PCR	High sensitivity Simple to use Rapid Cost-effective	Only species included in the PCR kit can be detected Multistep	[62]
California Mastitis Test	On-farm test Simple operation Fast	High subjectivity	[63]
Electrical conductivity test	Automated Fast Low cost	Results may be influenced by age or lactation period Relative low accuracy	[64]
Sensor	Good performance High sensitivity	High production technology requirements Affected by other diseases	[65]
IRT	Non-invasive Efficient	Affected by the surrounding environment	[66]

## 5. Milk Metabolomics

When cows develop mastitis, various metabolites in milk change, mainly as a result of the metabolic activities of pathogenic microorganisms, the secretions of white blood cells and damaged mammary epithelial cells, and the increased permeability of damaged mammary epithelial cells, with foreign substances passing directly from the blood to the milk [40]. More than 400 metabolites found in cow milk have been included in the livestock metabolome database (www.lmdb.ca, accessed on 4 April 2024) [67] and milk composition database (www.mcdb.ca, accessed on 4 April 2024) [68]. According to our statistical analysis, there is a total of 93 key differential metabolites (as shown in Table 2) in milk that can be used to distinguish cows with mastitis from healthy cows through the metabolomics technique and have profound effects on carbohydrate, energy, protein, and lipid metabolism.

The metabolomics of milk showed that carbohydrate metabolism was decreased in the milk of dairy cows with mastitis [69]. Due to the destruction of the udder, the permeability of the blood vessels in the mammary gland changes, resulting in an increase in Na^+^ and Cl^−^ in milk. In order to maintain the osmotic balance between the extracellular environment and milk, glucose is transported out of milk through the paracellular pathway. It is also possible that a decrease in blood flow to the mammary gland leads to a decrease in blood glucose supply and therefore a decrease in the concentration of glucose in CM milk samples [70]. Lactose is one of the main components of milk. Sundekilde et al. [71] conducted a metabolomics analysis of milk by NMR and found that the relative concentration of lactose in milk with a high SCC was low. Thomas et al. [69] conducted dynamic metabolomics studies through LC-MS. They challenged cows with *Streptococcus uberis*, and the lactose concentration in milk decreased over time. Lactose is the energy source of many bacteria, including *Streptococcus*. After a dairy cow is infected with mastitis, the related pathogens will consume a large amount of lactose, so that lactose cannot be detected in milk after 81 h from infection with *Streptococcus uberis* [69]. On the other hand, glucose is an important substance for lactose synthesis, and a reduction in glucose concentration due to mastitis affects lactose synthesis. In proteomic analysis, alpha-lactalbumin, the regulatory subunit of lactose synthase involved in lactose synthesis, was downregulated over time, supporting the decreasing trend of lactose concentration in the milk of cows with mastitis [72]. Short-chain fatty acids are straight or branched organic carboxylic acids produced by rumen microbial fermentation after ruminants eat carbohydrates, consisting of one to six carbon atoms [73]. Milk metabolomics showed that the concentration of short-chain fatty acids such as formate, acetate, acetoacetate, butyrate, β-hydroxybutyrate, and valerate was higher in milk samples of cows with mastitis [71,74,75]; it is shown in the literature that a negative energy balance in dairy cows leads to abnormal NEFA and β-hydroxybutyrate levels in the blood, resulting in suppressed immunity, elevated oxidative stress, and inflammatory changes, which are key indicators in dairy cows suffering from various diseases such as mastitis [76].

Milk metabolomics highlighted the importance of the TCA cycle for the health of dairy cows [77]. Infection causing mastitis leads to a decline in the overall regulation of this energy metabolism pathway. The levels of important intermediate metabolites in the TCA cycle, such as citrate, malate, cis-aconite, and ketoglutarate, were reduced in CM milk [75], and fumarate was less concentrated in high-SCC milk [71]. During mastitis, due to rumen wall damage, pathogenic bacteria will release a large amount of thiamine and endotoxin into the blood, promote the degradation of thiamine, inhibit its synthesis, lead to the downregulation of metabolite production in the TCA cycle, and reduce milk quality [50]. Studies have also shown that the levels of pyrophosphate, a precursor of high-energy phosphates and a major energy source for mitochondria, are significantly lower in the milk from cows with *Streptococcus agalactiae*-induced mastitis than in that from healthy cows, which may lead to lower mitochondrial energy allocation for milk synthesis in infected cows [78]. In addition, Hu et al. [35] discussed the potential of lactic acid, pyruvate, alanine, and succinic acid associated with the TCA cycle in predicting and treating mastitis. Among them, the levels of lactate, one of the main end products of bacterial carbohydrate metabolism, increased from 10 to 30 times after the development of SCM or CM, respectively. An increase in lactate concentration indicates increased bacterial metabolism [79] and is linearly correlated with the SCC [71], therefore being a valuable biomarker for the diagnosis of mastitis. With the increase in the number of somatic cells in milk, pyruvate metabolism increases, but alanine metabolism decreases. Succinic acid is an important starting material and intermediate product of the TCA cycle, and a change in its metabolism will disturb the TCA cycle; however, the effect of the metabolism of this substance on bovine mastitis still needs to be verified by further studies.

Changes in lipid metabolism in milk are mainly manifested in two metabolic pathways: glycerophospholipid metabolism and carnitine metabolism [80]. The lipid metabolism is downregulated with the aggravation of mastitis, and the bile metabolism also plays an important role in lipid metabolism [69]. D-glycerol 1-phosphate, also known as sn-glycerol 3-phosphate, is a glycerol phospholipid and a component of the cell membrane. It is significantly reduced in CM and SCM milk, which is mainly due to the destruction of the cell membrane of the mammary tissue and the action of phospholipase and carboxylesterase [80]. The decreased levels of sn-glycero-3-phosphocholine in milk samples of cows with mastitis also revealed the downregulation of the glycerophospholipid metabolism [50]. The results showed that the levels of L-carnitine and acylcarnitine were reduced in milk from cows with mastitis [80]. Carnitine plays an important role in fatty acid transport, transporting long-chain fatty acids from the cytosol to the mitochondrial matrix and influencing energy metabolism [81]. Acylcarnitine is an important participant in the fatty acid β-oxidation process, and its concentration changes reflect the level of fatty acid β-oxidation [82]. Thomas et al. [69] found that the levels of taurochenodeoxycholic acid, taurocholic acid, glycocholate, glycodeoxycholate, and cholate increased as a result of the effects of *Streptococcus* on milk. These bile acids promote antimicrobial and anti-inflammatory activity through the farnesoid X receptor pathway, inhibit the activation of the NF-κB signaling pathway, and reduce the levels of pro-inflammatory cytokines [39]. In addition, the studies of Tong et al. [78] and Wang et al. [50] showed that glycerol and ceramide (d18:1/22:0) in milk may also be potential biomarkers for the diagnosis of mastitis. The levels of ceramide (d18:1/22:0) in milk increased significantly when *Staphylococcus* and *Streptococcus* were the main pathogens, and that of glycerol increased significantly when *Streptococcus agalactiae* was the source of infection.

Due to the high activity of proteolytic enzymes such as plasmin, polymorphonuclear (PMN)-derived proteases, and bacterial proteases in milk, the content of total amino acids, especially branched-chain amino acids and aromatic amino acids, in milk with a high SCC was significantly increased [83]. The metabolomics of milk showed that Ala, Asp, and Glu metabolic pathways [74], Arg and Pro metabolic pathways [50], and Tyr metabolic pathways [75] are important. As for Tyr metabolism, 4-hydroxyphenylpyruvic acid can produce 4-hydroxyphenyllactic acid under the action of (R)-4-hydroxyphenyllactic dehydrogenase; the levels of 4-hydroxyphenylpyruvate and 4-hydroxyphenyllactic acid were reduced in the CM group compared to the healthy group, and that of 4-hydroxyphenyllactic acid was also significantly reduced in the CM group compared to the SCM group [75]. The association of Phe, catecholamines, and Tyr with thyroxin and catecholoestrogens may partly explain the differences in milk production and quality between cows with mastitis and healthy cows [78]. In addition, in the study of Sundekilde et al. [71], the content of Ile was higher in milk with a high SCC, and the concentration of hippurate produced by combining Gly and benzoate was lower in milk samples with a high SCC. In the study of Thomas et al. [69], the Leu content increased as *Streptococcus* mammary infection progressed. However, due to the limited role of the LC-MS technology in the identification of Leu, Ile, and other isomers, the role of Leu in the diagnosis of mastitis needs to be further verified by experiments. In addition to amino acids affected in mastitis, the levels of many small peptides were found to be increased in CM and SCM milk [80]. The increase in small peptides may be due to the activity of endogenous or bacterial-derived proteolytic enzymes or of both. On the other hand, it has also been suggested that small peptides are related to the SCC, because the SCC is associated with protease release, proteolytic activity, and the severity of mastitis [84]. Other studies showed that the metabolism of di-, tri-, and tetra-peptides in milk is upregulated after a *Streptococcus uberis* challenge [69].

**Table 2:** Summary of research on the prediction and diagnosis of mastitis in dairy cows using milk metabolomics.

Method	Type of Mastitis	Key Differential Metabolites	Important Metabolic Pathway	Reference
NMR	CM	Dimethylamine, Tyr, lactate, Leu, Pro, Val, Arg, Ile, fumarate, lactose, maltose, 2-oxoglutarate, citrate, cis-aconitate, creatine, O-acetylcarnitine, Gly, creatinine, carnitine, choline, N-acetylglucosamine, trimethylamine N-oxide	Metabolism: Gly, Ser, Thr; biosynthesis: Phe, Tyr, Trp; TCA cycle	[77]
NMR	SCM CM	SCM: hippurate, valerate, N-acetylglucosamine, His, Ile, Leu, fumarate CM: acetate, formate, lactate, benzoate, hippurate, β-hydroxybutyrate, valerate, N-acetylamino acid, Ala, His, Ile, Leu, Phe, Thr, Val	/	[74]
NMR	/	Lactate, acetate, β-hydroxybutyrate, butyrate, isoleucine, lactose, hippurate, fumarate	/	[71]
NMR	SCM CM	Lactate, Ala, pyruvate, succinate, formate, sn-glycero-3-phosphocholine	His metabolism; glycolysis; gluconeogenesis; lipolysis; β-oxidation; TCA cycle	[85]
GC-TOFMS	SCM	phenylpyruvic acid, homogentisic acid, 4-hydroxyphenylpyruvic acid, xanthine, guanine, uridine, glycerol, pyrophosphate	Metabolism: galactose, starch, sucrose, Ala, Asp, D-Glu, D-Gln; biosynthesis: Arg, neomycin, kanamycin, gentamicin; pentose and glucuronate interconversion; TCA cycle	[78]
LC-MS	SCM CM	2-Aminoethyl dihydrogen phosphate, 4,5-dihydroxyphthalic acid, O-succinyl-L-homoserine, isolithocholic acid, Tyr-Ile, dihydroxyacetone, D-fructofuranose, dichlorprop, fludrocortisone acetate, 2,4-dinitrotoluene	Metabolism: fructose, mannose, pyrimidine, 2-oxocarboxylic acid, nucleotides, glycerolipid, carbon; taste transduction; ABC transporters; bile secretion	[49]
LC-MS	SCM CM	Ceramide(d18:1/22:0), testosterone, glucuronide, 5-methyl-(THF), xanthine, 5-aminoimidazole ribonucleotide, thiamine, L-Arginine phosphate	Metabolism: sphingolipid, thiamine, purine, pyrimidine, glycerophospholipid, riboflavin, galactose, Cys, Met, Arg, Pro, Gly, Ser, Thr; biosynthesis: unsaturated fatty acids, steroid hormone; one-carbon pool by folate	[50]
LC-MS	/	Bile acids (taurochenodeoxycholic acid, taurocholic acid, glycocholate, glycodeoxycholate, cholate), hippurate, lactate, Leu, lactose, 2,3-dinor-8-iso PGF2α	Metabolism: Ala, Asp, Glu, pyrimidine, purine, ascorbate, aldarate, eicosanoids	[69]
LC-MS	CM	HODE, 13-HODE, 13-oxoODE, 9-HODE, 6-keto PGF_1α_, 11-HETE	Oxylipid biosynthesis	[86]

## 6. Blood Metabolomics

Blood is easy to collect and contains almost all of the body’s metabolites, giving a relatively complete picture of the body’s physiological and biochemical state [87]. Blood metabolomics can be divided into serum and plasma metabolomics, with the former being the most studied in recent years, because serum is considered the best biological fluid for discovering potential biomarkers to predict mastitis [88], and the signal strength of serum metabolites is higher than that of plasma metabolites when LC-MS is used [89,90]. The biomarkers used in blood metabolomics to predict and diagnose mastitis and key metabolic pathways are summarized in Table 3.

The most common methods for diagnosing SCM cows are CMT, SCC, and conductivity tests, but these tests are not used during the dry period and cannot predict the SCM status [91,92]. Therefore, the use of blood metabolomics to predict cows at risk of SCM during the dry period is essential for developing preventive measures and management strategies. Postpartum diagnosed SCM cows may have inflammation and mammary gland infection as early as 8 weeks prepartum. The systemic inflammatory response stimulates protein catabolism and provides substrates for the immune system to synthesize antibodies, cytokines, and acute-phase proteins, which may lead to a general increase in serum AA concentration. After mastitis was diagnosed, changes in AAs continued until 8 weeks postpartum, and Val, Ser, Tyr, and Phe had good predictive power for SCM at 8 and 4 weeks prenatal, with decreased levels in the serum of SCM cows [93]. In addition, Haxhiaj et al. [94] found that at 8 weeks and 4 weeks before parturition, there were four major changes in cows diagnosed with SCM compared with healthy cows. Firstly, the levels of branched-chain amino acids increased. Secondly, those of Ser and Gly decreased, which is inconsistent with the results of Dervishi et al. [93]. Thirdly, increased levels of glucose and trimethylamine-N-oxide (TMAO) were found. Fourthly, the levels of phosphatidylcholines, sphingomyelin, lysophosphatidylcholines, and acylcarnitines were mostly reduced. And studies have shown that five metabolites (Lys, Leu, Ile, kynurenine, and sphingomyelin C26:0) in serum at 8 weeks prepartum and 4 weeks prepartum in dairy cows can be used as predictive biomarkers of SCM [95]. Recent studies also looked for biomarkers that can predict the CM status during the dry period. 3’-sialyllactose is a kind of oligosaccharide that was proved to inhibit pathogenic microorganisms [96,97]. Zandkarimi et al. [98] showed that compared with healthy cows, the serum content of 3’-sialyllactose in CM cows during the perinatal period was significantly upregulated. N-methylethanolamine phosphate, choline, phosphorylcholine, free carnitine, trimethyl lysine, Tyr, and Pro can distinguish between CM and healthy cows at 3 and 2 weeks before parturition, and their levels are significantly upregulated.

In addition to help predict SCM during the dry period, blood metabolomics can also be used to screen diagnostic biomarkers. Some researchers injected *Escherichia coli* LPS into the udder of dairy cows, and the induced acute mastitis rapidly affected the secretion of plasma metabolites and cytokines, whose dynamic changes were associated with the development of clinical symptoms. However, the corresponding effects appeared late in milk [99]. Blood metabolomics shows greater potential than milk metabolomics in the early diagnosis of mastitis, and compared with milk metabolomics, which highlights the role of the TCA cycle, serum metabolomics highlights the role of the synthesis and degradation pathways of ketone bodies [77]. The decreased serum ketone body levels in cows with mastitis may be due to systemic metabolic changes caused by the local immune response to infection within the mammary gland [94]. Zhang et al. [95] identified five metabolites in the serum, including Lys, Leu, Ile, kynurenine, and sphingomyelin C26:1, as diagnostic markers of SCM through LC-MS/MS. Using GC-MS, the study showed that the combined detection of Val, Ile, Ser, and Pro can be used in the diagnosis of early SCM in lactation 4 to 8 weeks postpartum and that the levels of these AAs are significantly upregulated [93]. Qu [85] screened seven different metabolites (malonic acid, Phe, Ile, histamine, Leu, glucose, Gly) in plasma samples by NMR. With the increased amount of milk SCC, the metabolic levels of malonic acid and Gly in the plasma increased, while the metabolic levels of Phe, Ile, histamine, Leu, and glucose decreased. A control experiment was set up to explore the changes in serum metabolomics in the presence of intramammary infection caused by different pathogens. It was found that compared with the results for *Streptococcus agalactiae* infection, the levels of allantoin, which can be used to estimate rumen microbial protein production, and citrate, the intermediate metabolite of the TCA cycle, were lower only in the serum of dairy cows infected with *Prototheca* spp., and the level of Cit was higher only in the serum of *Prototheca* spp.-infected cows. However, most of the metabolites changed in the same way after intramammary infection, i.e., the levels of lactose, Val, His increased, and those of Asn, acetic acid, ethylenediamine decreased [100].

**Table 3:** Summary of research on the prediction and diagnosis of mastitis in dairy cows using blood metabolomics.

Method	Type of Mastitis	Key Differential Metabolites	Important Metabolic Pathway	Reference
NMR	SCM	His, lactose, acetate, Asn, dimethylamine, Val, Cit, methylguanidine, 3-hydroxybutyrate, acetone, allantoin, carnitine, citrate, ethanol	Ruminal fermentation, energy metabolism, urea synthesis and metabolism, immune and inflammatory response, mammary gland permeability	[100]
NMR	CM	Lactate, Ser, Phe, formate, Asn, citrate	/	[88]
NMR	SCM	His, lactose, acetate, Asp, dimethylamine, Val, carnitine, Cit, methylguanidine, 3-hydroxybutyrate, acetone, allantoin, ethanol, betaine, choline	Metabolism: Gly, Ser, Thr; biosynthesis: aminoacyl-tRNA, Phe, Tyr, Trp	[100]
NMR	SCM CM	Malonate, Phe, Ile, Histamine, Leu, glucose, Gly	His metabolism; glycolysis; gluconeogenesis; lipolysis; β-oxidation; citric acid cycle	[85]
NMR	CM	Lactose, Lactate, 3-hydroxybutyrate, acetoacetate, butyrate, acetate, citrate, propionate	/	[99]
GC-MS	SCM	Val, Ser, Tyr, Phe, Ile, Pro	Metabolism: propanoate, sphingolipid, Met, Phe, Tyr, Asp, Gly, Ser, Thr, porphyrin, glutathione; degradation: sphingolipid, Val, Leu, Ile; biosynthesis: protein, bile acid; ammonia recycling	[93]
LC-MS	SCM CM	20-Trihydroxy-leukotriene-B4, 13,14-dihydro-15-keto-PGE2, 9,10-dihydroxylinoleicacids, deoxycholic acid, 12-ketolithocholic acid, 3-hydroxyisovalerylcarnitine, citric acid, uric acid, inosine	Metabolism: arachidonic acid, linoleic acid, purine, carnitine, Ala, Asp, Glu; biosynthesis: primary bile acid, secondary bile acid; citrate cycle	[101]
LC-MS/MS	SCM	Leu, Ala, Orn, betaine, methylmalonate, lactate, pyruvate	Metabolism: Gly, Ser, Met, betaine, selenoamino acid; glucose–AlacCycle	[94]
LC-MS/MS	SCM	Kynurenine, sphingomyelin C26:0, Lys, Leu, Ile	Metabolism: biotin, Cys, Met, glutathione; degradation: Lys, Val, Leu, Ile; biosynthesis: Val, Leu, Ile, aminoacyl -tRNA	[95]
UPLC-MS^E^	CM	3′-Sialyllactose, N-methylethanolamine phosphate, choline, phosphorylcholine, free carnitine, trimethyl lysine, Tyr, Pro	Metabolism: carnitine, AA, water-soluble phospholipid	[98]

## 7. Milk Metabolomics and Blood Metabolomics Pathway Analysis

**Figure 2:**
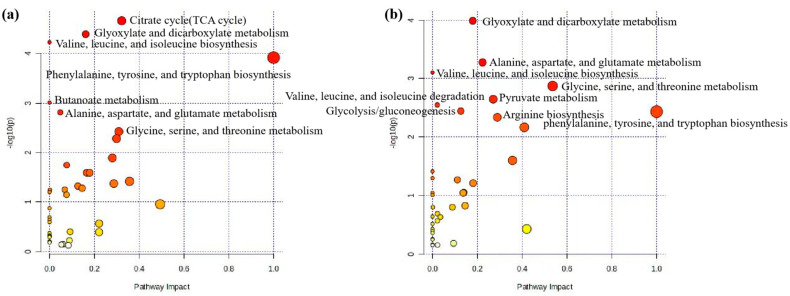
Pathway analysis of differential metabolites for prediction and early diagnosis of bovine mastitis. (**a**) Mastitis/healthy control metabolomics pathway analysis with milk samples, (**b**) mastitis/healthy control metabolomics pathway analysis with blood samples. Data were sourced from Supplementary Tables S1 and S2. Note: The sizes and colors of the circles represent the corresponding metabolites ratio and the log (p-value) of each pathway, respectively.

**Figure 3:**
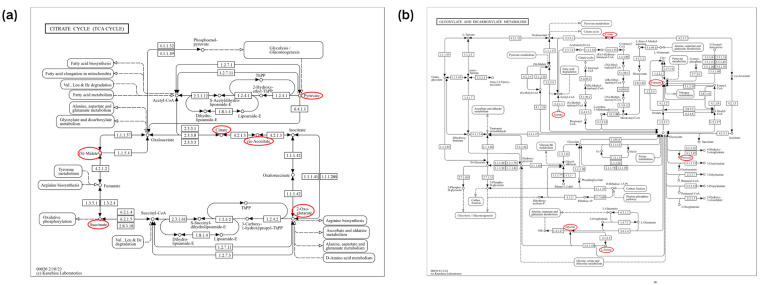
The mechanisms of the citrate cycle (TCA cycle), glyoxylate, and dicarboxylate metabolism pathways. The key differential metabolites associated with mastitis are circled in red. (**a**) The mechanisms of the citrate cycle (TCA cycle) pathway (bta00020). (**b**) The mechanisms of the glyoxylate and dicarboxylate metabolism pathway (bta00630). Sourced from the KEGG *Bos taurus* database.

In this section, we used MetaboAnalyst software (version 6.0) to analyze the metabolic pathways of 93 key differential metabolites reported in Table 2 [102,103]. The results showed that there were seven significant metabolic pathways (FDR < 0.05) (Figure 2a and Supplementary Table S1), namely, citrate cycle (TCA cycle); glyoxylate and dicarboxylate metabolism; valine, leucine, and isoleucine biosynthesis; valine, leucine, and isoleucine biosynthesis; phenylalanine, tyrosine, and tryptophan biosynthesis; butanoate metabolism; alanine, aspartate, and glutamate metabolism; and glycine, serine, and threonine metabolism. The most significant metabolic pathway was found to be the TCA cycle (FDR = 0.0015767), in which six metabolites (2-oxoglutarate, succinate, (s)-malate, cis-aconitate, citrate, pyruvate) were enriched, as shown in Figure 3a, reporting KEGG pathway analysis, TCA cycle, using the *Bos taurus* (cow) database (https://www.genome.jp/kegg/, accessed on 12 April 2024).

Similar to the pathway analysis of milk metabolomics, we applied MetaboAnalyst software (version 6.0) to analyze the metabolic pathways of 57 key differential metabolites in Table 3 [102,103]. The results showed that there were eight significant metabolic pathways (FDR < 0.05) (Figure 2b and Supplementary Table S2), namely, glyoxylate and dicarboxylate metabolism; alanine, aspartate, and glutamate metabolism; valine, leucine, and isoleucine biosynthesis; glycine, serine, and threonine metabolism; pyruvate metabolism; valine, leucine, and isoleucine degradation; glycolysis/gluconeogenesis; phenylalanine, tyrosine, and tryptophan biosynthesis; arginine biosynthesis. The most significant metabolic pathways were glyoxylate and dicarboxylate metabolism (FDR = 0.0081994), as six metabolites (citrate, l-serine, glycine, acetate, pyruvate and formate) were concentrated in this pathway, as shown in Figure 3b, reporting KEGG pathway analysis, glyoxylate and dicarboxylate metabolism, using the *Bos taurus* (cow) database (https://www.genome.jp/kegg/, accessed on 12 April 2024).

## 8. Metabolomics in Other Sample Types

In addition to the two biological fluids, milk and blood, used as the main samples for metabonomic studies, urine, rumen fluid, feces, tissues, and cells have also been used in recent years to study the prediction or early diagnosis of mastitis in dairy cows. This has helped us to find more comprehensive biomarkers of bovine mastitis. The key differential metabolites and metabolic pathways in these types of samples are listed in Table 4.

There were significant differences in the distribution of metabolites under different mammary gland health conditions. Zhu et al. [88] found through feces metabolomics that in dairy cows with mastitis, the levels of metabolites related to energy metabolism increased, such as propionate, acetate, pyruvate, acetoacetate, 2,3-butanediol, and ethanol, while the concentrations of Gly, acetylcholine, Gln, and O-phosphocholine decreased. In addition, the study found some similarities between feces and serum metabolites of cows with mastitis; SCM and CM cows had higher levels of pro-inflammatory lipid products (20-trihydroxy-leukotriene-B4, 13,14-dihydro-15-keto-PGE2, 9,10-dihydroxylinoleic acids) and lower levels of metabolites involved in secondary bile acid metabolism (deoxycholic acid, 12-ketolithocholic acid), energy metabolism (3-hydroxyisovalerylcarnitine, citric acid), and purine metabolism (uric acid and inosine) [101].

Compared with fecal extracts and serum, the metabolic spectrum of urine from cows with mastitis showed the greatest changes [88]. Cow urine is easy to collect and can be used to monitor cows for SCM during the dry period and two months postpartum [104]. There are few studies on the prediction or early diagnosis of mastitis using urine, but as one of the important substances to detect the health status of cows, it can be used to detect white blood cells, pH, ketone body concentration, and other indicators to judge the possible disease risk of cows [35]. A typical type of urine metabolism appeared to precede SCM and occurred 8 weeks before parturition. It was still present during the week of SCM diagnosis and up to 8 weeks postpartum. At 8 and 4 weeks prepartum, 24 and 27 metabolites distinguished SCM and CON cows, respectively. During the SCM diagnosis week, 22 metabolites differentiated SCM from CON cows. At 4 and 8 weeks postpartum, there were 13 and 28 metabolites that could distinguish the two types of cows, respectively. Among them, the levels of tetradecenoyl-L-carnitine, octadecadienyl-L-carnitine, Asp, carnosine, Glu, carnosine, Thr, SDMA (symmetric dimethylarginine), and glycerophospholipids (PC aa C34:2, PC ae C42:1) increased at 8 and 4 weeks prepartum and in the week of disease diagnosis and have value as predictive biomarkers [104].

The concentration of ethanol, dimethylamine, and acetate in the serum of SCM dairy cows decreased, as the products of rumen microbial metabolism, which could be due to impaired rumen fermentation [100]. Short-chain fatty acids are not only the energy source of dairy cows, but also the precursors of lactose, milk fat, and proteins. It was found that the composition of dairy milk is indirectly derived from the fermentation products of rumen microorganisms, and the rumen environment has a decisive influence on milk quality [105]. Therefore, the rumen, one of the main organs of dairy cows, is of great value in the study of bovine mastitis and of the improvement of milk quality. Rumen microbiome and metabolomics studies showed that the rumen microbiota, inflammation, and immune response-related metabolites underwent significant changes during CM. In the rumen of SCM-affected dairy cows, the relative abundance of several opportunistic pathogens and the levels of metabolites capable of producing antimicrobial compounds or having competitive inhibitory effects were increased. During intramammary infections (IMIs), short-chain fatty acid (SCFA)-producing bacteria and probiotics decreased in the rumen, which was accompanied by a decrease in 2-phenylbutyric acid; in addition, research shows that the levels of 12-oxo-20-dihydroxy-leukotriene B4 and 10beta-hydroxy-6beta-isobutyrylfuranoeremophilane in the rumen of CM cows and 6-methoxymellein and 5-hydroxymethyl-2-furancarboxaldehyde (5-HMF) in the rumen of SCM cows significantly increased, indicating their high application value as biomarkers of mastitis in dairy cows [106].

Tissues can also be used as samples for metabolomics studies [107]. Ryman et al. [86] combined milk and mammary metabolomics and found that the levels of oxylipids derived from arachidonic acid and linoleic acid were significantly increased in the mammary gland of *Streptococcus uberis*-infected cows.

**Table 4:** Summary of research on the prediction and diagnosis of mastitis in dairy cows using metabolomics in various sample types.

Sample Type	Method	Type of Mastitis	Key Differential Metabolites	Important Metabolic Pathway	Reference
mammary gland	LC-MS	CM	6-Keto prostaglandin F1α, prostaglandin E2, prostaglandin F2α, 5-oxoeicosatetraenoic acid, 9-hydroxyoctadecadienoic acid, 13-hydroxyoctadecadienoic acid, 9-oxooctadecadienoic acid	Oxylipid biosynthesis	[86]
urine	NMR	CM	Citrate, lactose, taurine, methylamine, N-acetylaspartate, galactose, 3-phenylpropionate	/	[88]
	DI/LC-MS/MS	SCM	acylcarnitines (C3:1, C3-OH, C5-DC (C6-OH), C5-M-DC, C14:1, C18:2, C12, C14, C16:2), phosphatidylcholines (PC aa C34:2, PC ae C42:1, PC ae C36:4), AA (Asp, Glu, His, Thr), biogenic amines (carnosine, symmetric dimethylarginine), hexose	Metabolism: selenoamino acid, Asp, arachidonic acid, Cys, Tyr, Met, sphingolipid; biosynthesis: phosphatidylethanolamine, fatty acids; ammonia recycling; malate–aspartate shuttle; homocysteine degradation	[104]
feces	NMR	CM	Propionate, acetate, pyruvate, acetoacetate, 2,3-butanediol, ethanol, glycine, O-acetylcholine, glutamine, O-phosphocholine,	/	[88]
	LC-MS	SCM CM	20-trihydroxy-leukotriene-B4, 13,14-dihydro-15-keto-PGE2, 9,10-dihydroxylinoleicacids, deoxycholic acid, 12-ketolithocholic acid, 3-hydroxyisovalerylcarnitine, citric acid, uric acid, inosine	Metabolism: arachidonic acid, linoleic acid, purine, carnitine, Ala, Asp, Glu; biosynthesis: primary bile acid, secondary bile acid; citrate cycle	[101]
rumen fluid	LC-MS/MS	SCM CM	SCM: methenamine, 5-hydroxymethyl-2-furancarboxaldehyde, 6-methoxymellein CM: 12-oxo-20-dihydroxy-leukotriene B4, 10beta-hydroxy-6beta-isobutyrylfuranoeremophilane, 2-phenylbutyric acid	Metabolism: arachidonic acid, butanoate; degradation: limonene, pinene, furfural; biosynthesis: formaldehyde, plant secondary metabolites	[106]

## 9. Existing Knowledge Gap and Future Recommendations

Due to differences in metabolomics platforms, methods, and sample sizes across various studies, there is a disparity in the biomarkers identified. Moreover, biomarkers determined solely by metabolomics cannot fully represent biological changes, as they require large-scale cohort validation and clinical verification processes to serve as indicators of biochemical changes in the body. Therefore, the selection and application of biomarkers is a task of practical significance but also poses considerable challenges. To enhance the diagnostic power of metabolomics biomarkers, extensive research is needed to establish standardized analytical processes for validation. In the future, the combined analyses of metabolomics and other omics technologies as well as multi-sample analysis will play a huge scientific research role. Furthermore, existing metabolomics technologies are not only used for screening biomarkers of bovine mastitis for early diagnosis and prediction, but also widely applied in studying the therapeutic effects of certain plant extracts or drugs on mastitis. These types of studies often utilize mouse models, and the efficacy of these substances can be further verified in dairy cows.

## 10. Conclusions

Metabolomics, the most downstream aspect of gene expression, can amplify the subtle changes in gene and protein expression at the metabolite level. The metabolomics technique combined with multivariate statistical analysis can display the metabolic profile of dairy cows in a panoramic view and screen biomarkers for the prediction and early diagnosis of mastitis. With the development of metabolome detection and analysis platforms, this technology will give us a deeper understanding of bovine mastitis, allowing for its prevention and early treatment, and reduce its harm to the dairy farming industry and human food safety. This review summarizes the pathogenesis, the diagnostic methods of bovine mastitis, and the application of the metabolomic technology in the early diagnosis and prediction of bovine mastitis. It also provides new perspectives for the prevention and treatment of mastitis by analyzing small-molecule metabolites in blood and milk.

## Data Availability

All data generated or analyzed during this study are included in this published article (and its Supplementary Information files).

## Supplementary Materials

The following supporting information can be downloaded at: https://www.mdpi.com/article/10.3390/ani14152264/s1, Table S1: The pathway enrichment results of differential metabolites in milk; Table S2: The pathway enrichment results of differential metabolites in blood.

## References

[1] Hogeveen H., Steeneveld W., Wolf C.A. (2019). Production diseases reduce the efficiency of dairy production: A review of the results, methods, and approaches regarding the economics of mastitis. Annu. Rev. Resour. Econ..

[2] Tommasoni C., Fiore E., Lisuzzo A., Gianesella M. (2023). Mastitis in dairy cattle: On-Farm diagnostics and future perspectives. Animals.

[3] Laliotis G.P., Koutsouli P., Sotirakoglou K., Savoini G., Politis I. (2020). Association of Oxidative Stress Biomarkers and Clinical Mastitis Incidence in Dairy Cows During the Periparturient Period. J. Vet. Res..

[4] Cobirka M., Tancin V., Slama P. (2020). Epidemiology and classification of mastitis. Animals.

[5] Suzuki N., Kurose T., Kaneko S., Haraguchi A., Isobe N. (2020). Outcome prediction from the first examination in clinical mastitis using ultrasonography in dairy cows. Anim. Sci. J..

[6] Shaheen T., Ahmad S.B., Rehman M.U., Muzamil S., Bhat R.R., Hussain I., Bashir N., Mir M.U.R., Paray B.A., Dawood M.A.O. (2020). Investigations on cytokines and proteins in lactating cows with and without naturally occurring mastitis. J. King Saud. Univ. Sci..

[7] Rienesl L., Khayatzdadeh N., Koeck A., Egger-Danner C., Gengler N., Grelet C., Dale L.M., Werner A., Auer F., Leblois J. (2022). Prediction of Acute and Chronic Mastitis in Dairy Cows Based on Somatic Cell Score and Mid-Infrared Spectroscopy of Milk. Animals.

[8] Li X., Xu C., Liang B., Kastelic J.P., Han B., Tong X., Gao J. (2023). Alternatives to antibiotics for treatment of mastitis in dairy cows. Front. Vet. Sci..

[9] Rollin E., Dhuyvetter K.C., Overton M.W. (2015). The cost of clinical mastitis in the first 30 days of lactation: An economic modeling tool. Prev. Vet. Med..

[10] Halasa T., Huijps K., Osteras O., Hogeveen H. (2007). Economic effects of bovine mastitis and mastitis management: A review. Vet. Q..

[11] Zhao X., Lacasse P. (2008). Mammary tissue damage during bovine mastitis: Causes and control. J. Anim. Sci..

[12] Blum S.E., Heller E.D., Leitner G. (2014). Long term effects of *Escherichia coli* mastitis. Vet. J..

[13] Aghamohammadi M., Haine D., Kelton D.F., Barkema H.W., Hogeveen H., Keefe G.P., Dufour S. (2018). Herd-Level mastitis-associated costs on canadian dairy farms. Front. Vet. Sci..

[14] Mbindyo C.M., Gitao G.C., Mulei C.M. (2020). Prevalence, etiology, and risk factors of mastitis in dairy cattle in embu and kajiado counties, kenya. Vet. Med. Int..

[15] Califf R.M. (2018). Biomarker definitions and their applications. Exp. Biol. Med..

[16] Chen X.X., Xu Y.M., Lau A. (2022). Metabolic effects of long-term cadmium exposure: An overview. Environ. Sci. Pollut. Res..

[17] Porto V.A., Da R.J.E., Ursulino J.S., Porto R.S., Da S.M., de Jesus L., Oliveira J.M., Crispim A.C., Santos J., Aquino T.M. (2023). NMR-based metabolomics applied to ecotoxicology with zebrafish (*Danio rerio*) as a prominent model for metabolic profiling and biomarker discovery: Overviewing the most recent approaches. Sci. Total Environ..

[18] Sille F., Hartung T. (2024). Metabolomics in preclinical drug safety assessment: Current status and future trends. Metabolites.

[19] Yu J.W., Song M.H., Lee J.H., Song J.H., Hahn W.H., Keum Y.S., Kang N.M. (2024). Urinary metabolomic differentiation of infants fed on human breast milk and formulated milk. Metabolites.

[20] Gao Y., Hou L., Gao J., Li D., Tian Z., Fan B., Wang F., Li S. (2021). Metabolomics approaches for the comprehensive evaluation of fermented foods: A review. Foods.

[21] Legrand E., Basu N., Hecker M., Crump D., Xia J., Chandramouli B., Butler H., Head J.A. (2021). Targeted metabolomics to assess exposure to environmental chemicals of concern in japanese quail at two life stages. Metabolites.

[22] Wishart D.S., Cheng L.L., Copie V., Edison A.S., Eghbalnia H.R., Hoch J.C., Gouveia G.J., Pathmasiri W., Powers R., Schock T.B. (2022). NMR and metabolomics—A roadmap for the future. Metabolites.

[23] Monteiro M.S., Carvalho M., Bastos M.L., Guedes D.P.P. (2013). Metabolomics analysis for biomarker discovery: Advances and challenges. Curr. Med. Chem..

[24] Eriksson Å., Persson Waller K., Svennersten-Sjaunja K., Haugen J., Lundby F., Lind O. (2005). Detection of mastitic milk using a gas-sensor array system (electronic nose). Int. Dairy J..

[25] Sadat A., Farag A.M.M., Elhanafi D., Awad A., Elmahallawy E.K., Alsowayeh N., El-khadragy M.F., Elshopakey G.E. (2023). Immunological and Oxidative Biomarkers in Bovine Serum from Healthy, Clinical, and Sub-Clinical Mastitis Caused by *Escherichia coli* and *Staphylococcus aureus* Infection. Animals.

[26] Campos B., Pickering A.C., Rocha L.S., Aguilar A.P., Fabres-Klein M.H., de Oliveira M.T., Fitzgerald J.R., de Oliveira B.R.A. (2022). Diversity and pathogenesis of *Staphylococcus aureus* from bovine mastitis: Current understanding and future perspectives. BMC Vet. Res..

[27] Benic M., Macesic N., Cvetnic L., Habrun B., Cvetnic Z., Turk R., Duricic D., Lojkic M., Dobranic V., Valpotic H. (2018). Bovine mastitis: A persistent and evolving problem requiring novel approaches for its control—A review. Vet. Arh..

[28] Aitken S.L., Corl C.M., Sordillo L.M. (2011). Immunopathology of mastitis: Insights into disease recognition and resolution. J. Mammary Gland. Biol. Neoplasia.

[29] Girma A., Tamir D. (2022). Prevalence of Bovine Mastitis and its associated risk factors among dairy cows in ethiopia during 2005-2022: A systematic review and meta-analysis. Vet. Med. Int..

[30] Cheng W.N., Han S.G. (2020). Bovine mastitis: Risk factors, therapeutic strategies, and alternative treatments—A review. Asian Australas. J. Anim. Sci..

[31] Khan M.Z., Khan A., Xiao J., Ma J., Ma Y., Chen T., Shao D., Cao Z. (2020). Overview of research development on the role of nf-kappab signaling in mastitis. Animals.

[32] Taponen S., Pyorala S. (2009). Coagulase-negative *staphylococci* as cause of bovine mastitis—Not so different from *Staphylococcus aureus*?. Vet. Microbiol..

[33] Wu F., Xie X., Du T., Jiang X., Miao W., Wang T. (2023). *Lactococcus lactis*, a bacterium with probiotic functions and pathogenicity. World J. Microbiol. Biotechnol..

[34] Song J., Xiang W., Wang Q., Yin J., Tian T., Yang Q., Zhang M., Ge G., Li J., Diao N. (2023). Prevalence and risk factors of *Klebsiella* spp. in milk samples from dairy cows with mastitis-A global systematic review. Front. Vet. Sci..

[35] Hu H., Fang Z., Mu T., Wang Z., Ma Y., Ma Y. (2021). Application of metabolomics in diagnosis of cow mastitis: A review. Front. Vet. Sci..

[36] Bao L., Sun H., Zhao Y., Feng L., Wu K., Shang S., Xu J., Shan R., Duan S., Qiu M. (2023). Hexadecanamide alleviates *Staphylococcus aureus*-induced mastitis in mice by inhibiting inflammatory responses and restoring blood-milk barrier integrity. PLoS Pathog..

[37] Müller U., Neu-Zahren A., Sauerwein H. (2006). Milking-induced changes of the teat canal: Review of investigation methods and first results from testing teat canal penetrability. Milchwissenschaft.

[38] Rainard P., Riollet C. (2006). Innate immunity of the bovine mammary gland. Vet. Res..

[39] Haxhiaj K., Wishart D.S., Ametaj B.N. (2022). Mastitis: What it is, current diagnostics, and the potential of metabolomics to dentify new predictive biomarkers. Dairy.

[40] Hettinga K.A., van Valenberg H.J., Lam T.J., van Hooijdonk A.C. (2008). Detection of mastitis pathogens by analysis of volatile bacterial metabolites. J. Dairy. Sci..

[41] Kelsey J.A., Bayles K.W., Shafii B., Mcguire M.A. (2006). Fatty acids and monoacylglycerols inhibit growth of *Staphylococcus aureus*. Lipids.

[42] Bhattarai D., Worku T., Dad R., Rehman Z.U., Gong X., Zhang S. (2018). Mechanism of pattern recognition receptors (PRRs) and host pathogen interplay in bovine mastitis. Microb. Pathog..

[43] Turk R., Koledic M., Macesic N., Benic M., Dobranic V., Duricic D., Cvetnic L., Samardzija M. (2017). The role of oxidative stress and inflammatory response in the pathogenesis of mastitis in dairy cows. Mljekarstvo.

[44] Zhang K., Zhang M., Su H., Zhao F., Wang D., Zhang Y., Cao G., Zhang Y. (2024). Regulation of Inflammatory Responses of Cow Mammary Epithelial Cells through MAPK Signaling Pathways of IL-17A Cytokines. Animals.

[45] Sordillo L.M., Streicher K.L. (2002). Mammary gland immunity and mastitis susceptibility. J. Mammary Gland. Biol. Neoplasia.

[46] Ezzat A.M., Quintela-Baluja M., Bohme K., Fernandez-No I., Caamano-Antelo S., Calo-Mata P., Barros-Velazquez J. (2014). The immunology of mammary gland of dairy ruminants between healthy and inflammatory conditions. J. Vet. Med..

[47] Damm M., Holm C., Blaabjerg M., Bro M.N., Schwarz D. (2017). Differential somatic cell count-A novel method for routine mastitis screening in the frame of Dairy Herd Improvement testing programs. J. Dairy. Sci..

[48] Wittek T., Mader C., Ribitsch V., Burgstaller J. (2019). Measurement of oxygen concentration for detection of subclinical mastitis. Schweiz. Arch. Tierheilkd..

[49] She Y., Liu J., Su M., Li Y., Guo Y., Liu G., Deng M., Qin H., Sun B., Guo J. (2023). A study on differential biomarkers in the milk of holstein cows with different somatic cells count levels. Animals.

[50] Wang Y., Nan X., Zhao Y., Wang H., Wang M., Jiang L., Zhang F., Xue F., Hua D., Li K. (2020). Coupling 16S rDNA sequencing and untargeted mass spectrometry for milk microbial composition and metabolites from dairy cows with clinical and subclinical mastitis. J. Agric. Food. Chem..

[51] Saila S., Bork O., Tucker I.G., Cranefield S., Bryan M.A. (2023). Evaluation of an on-farm culture system for the detection of subclinical mastitis pathogens in dairy cattle. JDS Commun..

[52] Mansion-De V.E., Knorr N., Paduch J.H., Zinke C., Hoedemaker M., Kromker V. (2014). A field study evaluation of petrifilm plates as a 24-h rapid diagnostic test for clinical mastitis on a dairy farm. Prev. Vet. Med..

[53] Kahya D.S., Yildiz M., Akkoc A., Mutlu A.M., Ardicli O., Aner H. (2024). Comparison of bacteriological culture method and multiplex real-time PCR for detection of mastitis. Res. Vet. Sci..

[54] Bhutto A.L., Murray R.D., Woldehiwet Z. (2012). California mastitis test scores as indicators of subclinical intra-mammary infections at the end of lactation in dairy cows. Res. Vet. Sci..

[55] Wollowski L., Bertulat S., Kossatz A., Heuwieser W. (2019). Short communication: Diagnosis and classification of clinical and subclinical mastitis utilizing a dynamometer and a handheld infrared thermometer. J. Dairy Sci..

[56] Mota-Rojas D., Pereira A.M.F., Wang D., Martínez-Burnes J., Ghezzi M., Hernández-Avalos I., Lendez P., Mora-Medina P., Casas A., Olmos-Hernández A. (2021). Clinical Applications and Factors Involved in Validating Thermal Windows Used in Infrared Thermography in Cattle and River Buffalo to Assess Health and Productivity. Animals.

[57] Carvalho-Sombra T., Fernandes D.D., Bezerra B., Nunes-Pinheiro D. (2021). Systemic inflammatory biomarkers and somatic cell count in dairy cows with subclinical mastitis. Vet. Anim. Sci..

[58] Usui M., Akiyoshi M., Fukuda A., Iwano H., Kato T. (2023). 16S rRNA nanopore sequencing for rapid diagnosis of causative bacteria in bovine mastitis. Res. Vet. Sci..

[59] Schukken Y.H., Wilson D.J., Welcome F., Garrison-Tikofsky L., Gonzalez R.N. (2003). Monitoring udder health and milk quality using somatic cell counts. Vet. Res..

[60] Ruegg P.L., Pantoja J.C.E. (2013). Understanding and using somatic cell counts to improve milk quality. Ir. J. Agric. Food Res..

[61] Ashraf A., Imran M. (2018). Diagnosis of bovine mastitis: From laboratory to farm. Trop. Anim. Health Prod..

[62] Soltau J.B., Einax E., Klengel K., Katholm J., Failing K., Wehrend A., Donat K. (2017). Within-herd prevalence thresholds for herd-level detection of mastitis pathogens using multiplex real-time PCR in bulk tank milk samples. J. Dairy. Sci..

[63] Adkins P.R.F., Middleton J.R. (2018). Methods for diagnosing mastitis. Vet. Clin. N. Am. Food. Anim. Pract..

[64] Pyoeraelae S.U.O.H. (2003). Indicators of inflammation in the diagnosis of mastitis. Vet. Res..

[65] Hogeveen H., Klaas I.C., Dalen G., Honig H., Zecconi A., Kelton D.F., Sanchez M.M. (2021). Novel ways to use sensor data to improve mastitis management. J. Dairy. Sci..

[66] Colak A., Polat B., Okumus Z., Kaya M., Yanmaz L.E., Hayirli A. (2008). Short communication: Early detection of mastitis using infrared thermography in dairy cows. J. Dairy. Sci..

[67] Goldansaz S.A., Guo A.C., Sajed T., Steele M.A., Plastow G.S., Wishart D.S. (2017). Livestock metabolomics and the livestock metabolome: A systematic review. PLoS ONE.

[68] Foroutan A., Guo A.C., Vazquez-Fresno R., Lipfert M., Zhang L., Zheng J., Badran H., Budinski Z., Mandal R., Ametaj B.N. (2019). Chemical composition of commercial cow’s milk. Agric. Food. Chem..

[69] Thomas F.C., Mudaliar M., Tassi R., Mcneilly T.N., Burchmore R., Burgess K., Herzyk P., Zadoks R.N., Eckersall P.D. (2016). Mastitomics, the integrated omics of bovine milk in an experimental model of *Streptococcus uberis* mastitis: 3. Untargeted metabolomics. Mol. Biosyst..

[70] Bansal B.K., Hamann J., Grabowskit N.T., Singh K.B. (2005). Variation in the composition of selected milk fraction samples from healthy and mastitic quarters, and its significance for mastitis diagnosis. J. Dairy. Res..

[71] Sundekilde U.K., Poulsen N.A., Larsen L.B., Bertram H.C. (2013). Nuclear magnetic resonance metabonomics reveals strong association between milk metabolites and somatic cell count in bovine milk. J. Dairy. Sci..

[72] Mudaliar M., Tassi R., Thomas F.C., Mcneilly T.N., Weidt S.K., Mclaughlin M., Wilson D., Burchmore R., Herzyk P., Eckersall P.D. (2016). Mastitomics, the integrated omics of bovine milk in an experimental model of *Streptococcus uberis* mastitis: 2. Label-free relative quantitative proteomics. Mol. Biosyst..

[73] Rios-Covian D., Ruas-Madiedo P., Margolles A., Gueimonde M., de Los Reyes-Gavilan C.G., Salazar N. (2016). Intestinal short Chain fatty acids and their link with diet and human health. Front. Microbiol..

[74] Luangwilai M., Duangmal K., Chantaprasarn N., Settachaimongkon S. (2021). Comparative metabolite profiling of raw milk from subclinical and clinical mastitis cows using ^1^H-NMR combined with chemometric analysis. Int. J. Food Sci. Technol..

[75] Xi X., Kwok L.Y., Wang Y., Ma C., Mi Z., Zhang H. (2017). Ultra-performance liquid chromatography-quadrupole-time of flight mass spectrometry MS^E^-based untargeted milk metabolomics in dairy cows with subclinical or clinical mastitis. J. Dairy. Sci..

[76] Huang B., Khan M.Z., Kou X., Chen Y., Liang H., Ullah Q., Khan N., Khan A., Chai W., Wang C. (2023). Enhancing metabolism and milk production performance in periparturient dairy cattle through rumen-protected methionine and choline supplementation. Metabolites.

[77] Zhu C., Tang K., Lu X., Tang J., Laghi L. (2021). An untargeted metabolomics investigation of milk from dairy cows with clinical mastitis by ^1^H-NMR. Foods.

[78] Tong J., Zhang H., Zhang Y., Xiong B., Jiang L. (2019). Microbiome and metabolome analyses of milk from dairy cows with subclinical *streptococcus agalactiae* mastitis-potential biomarkers. Front. Microbiol..

[79] Davis S.R., Farr V.C., Prosser C.G., Nicholas G.D., Turner S.A., Lee J., Hart A.L. (2004). Milk L-lactate concentration is increased during mastitis. J. Dairy Res..

[80] Xi X.M. (2016). Microbial Diversity and Metabolomics Studies on Milk during Bovine Mastitis. Doctoral Thesis.

[81] Sundekilde U.K., Frederiksen P.D., Clausen M.R., Larsen L.B., Bertram H.C. (2011). Relationship between the Metabolite Profile and Technological Properties of Bovine Milk from Two Dairy Breeds Elucidated by NMR-Based Metabolomics. J. Agric. Food Chem..

[82] Zhou L., Wang Q., Yin P., Xing W., Wu Z., Chen S., Lu X., Zhang Y., Lin X., Xu G. (2012). Serum metabolomics reveals the deregulation of fatty acids metabolism in hepatocellular carcinoma and chronic liver diseases. Anal. Bioanal. Chem..

[83] Caggiano N., Smirnoff A.L., Bottini J.M., De Simone E.A. (2019). Protease activity and protein profile in milk from healthy dairy cows and cows with different types of mastitis. Int. Dairy J..

[84] Wedholm A., Moller H.S., Lindmark-Mansson H., Rasmussen M.D., Andren A., Larsen L.B. (2008). Identification of peptides in milk as a result of proteolysis at different levels of somatic cell counts using LC MALDI MS/MS detection. J. Dairy Res..

[85] Qu K.C. (2020). ^1^H nuclear Magnetic Resonance-Based Metabonomics on Early-Stage Diagnosis of Dairy Cow Mastitis. Doctoral Thesis.

[86] Ryman V.E., Pighetti G.M., Lippolis J.D., Gandy J.C., Applegate C.M., Sordillo L.M. (2015). Quantification of bovine oxylipids during intramammary *Streptococcus uberis* infection. Prostaglandins Other Lipid Mediat..

[87] Demicheva E., Dordiuk V., Espino F.P., Ushenin K., Aboushanab S., Shevyrin V., Buhler A., Mukhlynina E., Solovyova O., Danilova I. (2024). Advances in mass spectrometry-based blood metabolomics profiling for non-cancer diseases: A comprehensive review. Metabolites.

[88] Zhu C., Zhang Q., Zhao X., Yang Z., Yang F., Yang Y., Tang J., Laghi L. (2023). Metabolomic analysis of multiple biological specimens (feces, serum, and urine) by (1)H-NMR spectroscopy from dairy cows with clinical mastitis. Animals.

[89] Lin Z., Zhang Z., Lu H., Jin Y., Yi L., Liang Y. (2014). Joint MS-based platforms for comprehensive comparison of rat plasma and serum metabolic profiling. Biomed. Chromatogr..

[90] Yu Z., Kastenmuller G., He Y., Belcredi P., Moller G., Prehn C., Mendes J., Wahl S., Roemisch-Margl W., Ceglarek U. (2011). Differences between human plasma and serum metabolite profiles. PLoS ONE.

[91] Hurley W.L., Theil P.K. (2011). Perspectives on immunoglobulins in colostrum and milk. Nutrients.

[92] Khatun M., Clark C.E.F., Lyons N.A., Thomson P.C., Kerrisk K.L., Garcia S.C. (2017). Early detection of clinical mastitis from electrical conductivity data in an automatic milking system. Anim. Prod. Sci..

[93] Dervishi E., Zhang G., Dunn S.M., Mandal R., Wishart D.S., Ametaj B.N. (2017). GC-MS metabolomics identifies metabolite alterations that precede subclinical mastitis in the blood of transition dairy cows. J. Proteome Res..

[94] Haxhiaj K., Li Z., Johnson M., Dunn S.M., Wishart D.S., Ametaj B.N. (2022). Blood metabolomic phenotyping of dry cows could predict the high milk somatic cells in early lactation—Preliminary results. Dairy.

[95] Zhang G., Tobolski D., Zwierzchowski G., Mandal R., Wishart D.S., Ametaj B.N. (2022). Identification of serum-predictive biomarkers for subclinical mastitis in dairy cows and new insights into the pathobiology of the disease. J. Agric. Food. Chem..

[96] Nakamura T., Kawase H., Kimura K., Watanabe Y., Ohtani M., Arai I., Urashima T. (2003). Concentrations of sialyloligosaccharides in bovine colostrum and milk during the prepartum and early lactation. J. Dairy. Sci..

[97] Ten B.S., Bovee-Oudenhoven I.M., Feitsma A.L., van Hoffen E., Schoterman M.H. (2014). Functional role and mechanisms of sialyllactose and other sialylated milk oligosaccharides. Nutr. Rev..

[98] Zandkarimi F., Vanegas J., Fern X., Maier C.S., Bobe G. (2018). Metabotypes with elevated protein and lipid catabolism and inflammation precede clinical mastitis in prepartal transition dairy cows. J. Dairy. Sci..

[99] Johnzon C.F., Dahlberg J., Gustafson A.M., Waern I., Moazzami A.A., Ostensson K., Pejler G. (2018). The effect of lipopolysaccharide-induced experimental bovine mastitis on clinical parameters, inflammatory markers, and the metabolome: A kinetic approach. Front. Immunol..

[100] Lisuzzo A., Laghi L., Fiore E., Cecchinato A., Bisutti V., Pegolo S., Giannuzzi D., Tessari R., Barberio A., Schiavon E. (2024). Serum metabolome differences associated with subclinical intramammary infection caused by *Streptococcus agalactiae* and *Prototheca* spp. in multiparous dairy cows. J. Dairy. Sci..

[101] Wang Y., Nan X., Zhao Y., Jiang L., Wang H., Zhang F., Hua D., Liu J., Yang L., Yao J. (2022). Discrepancies among healthy, subclinical mastitic, and clinical mastitic cows in fecal microbiome and metabolome and serum metabolome. J. Dairy. Sci..

[102] Hao D., Bai J., Du J., Wu X., Thomsen B., Gao H., Su G., Wang X. (2021). Overview of metabolomic analysis and the integration with multi-omics for economic traits in cattle. Metabolites.

[103] Chen J., Amdanee N., Zuo X., Wang Y., Gong M., Yang Y., Li H., Zhang X., Zhang C. (2024). Biomarkers of bipolar disorder based on metabolomics: A systematic review. J. Affect. Disord..

[104] Zwierzchowski G., Zhang G., Mandal R., Wishart D.S., Ametaj B.N. (2020). Mass-spec-based urinary metabotyping around parturition identifies screening biomarkers for subclinical mastitis in dairy cows. Res. Vet. Sci..

[105] Lunsin R., Wanapat M., Rowlinson P. (2012). Effect of cassava hay and rice bran oil supplementation on rumen fermentation, milk yield and milk composition in lactating dairy cows. Asian Australas. J. Anim. Sci..

[106] Wang Y., Nan X., Zhao Y., Jiang L., Wang M., Wang H., Zhang F., Xue F., Hua D., Liu J. (2021). Rumen microbiome structure and metabolites activity in dairy cows with clinical and subclinical mastitis. J. Anim. Sci. Biotechnol..

[107] Zhu Y., Bu D., Ma L. (2022). Integration of multiplied omics, a step forward in systematic dairy research. Metabolites.

